# Predictors of early sexual initiation among a nationally representative sample of Nigerian adolescents

**DOI:** 10.1186/1471-2458-8-136

**Published:** 2008-04-25

**Authors:** Adesegun O Fatusi, Robert W Blum

**Affiliations:** 1 Department of Community Health, College of Health Sciences, Obafemi Awolowo University, Ile-Ife, Nigeria; 2 Department of Population, Family & Reproductive Health, Johns Hopkins Bloomberg School of Public Health, Baltimore, Maryland, USA

## Abstract

**Background:**

Early sexual debut among adolescents is associated with considerable negative heath and development outcomes. An understanding of the determinants or predictors of the timing of sexual debut is important for effective intervention, but very few studies to date have addressed this issue in the Nigerian context. The aim of the present study is to examine predictors of adolescent sexual initiation among a nationally representative sample of adolescents in Nigeria.

**Methods:**

Interviewer-collected data of 2,070 never-married adolescents aged 15–19 years were analysed to determine association between age of sexual debut and demographic, psychosocial and community factors. Using Cox proportional hazards regression multivariate analysis was carried out with two different models – one with and the other without psychosocial factors. Hazard ratio (HR) and 95% confidence interval (CI) were calculated separately for males and females.

**Results:**

A fifth of respondents (18% males; 22% females) were sexually experienced. In the South 24.3% males and 28.7% females had initiated sex compared to 12.1% of males and 13.1% females in the North (p < 0.001). In the first model, only region was significantly associated with adolescent sexual initiation among both males and females; however, educational attainment and age were also significant among males. In the second (psychosocial) model factors associated with adolescent sexual debut for both genders included more positive attitudes regarding condom efficacy (males: HR = 1.28, 95% CI = 1.07–1.53; females: HR = 1.24, 95% CI = 1.05–1.46) and more positive attitudes to family planning use (males: HR = 1.19, 95% CI = 1.09–1.31; females: HR = 1.18, 95% CI = 1.07–1.30). A greater perception of condom access (HR = 1.42, 95% CI = 1.14–1.76) and alcohol use (HR = 1.90, 95% CI = 1.38–2.62) among males and positive gender-related attitudes (HR = 1.13, 95% CI = 1.04–1.23) among females were also associated with increased likelihood of adolescent sexual initiation. Conversely, personal attitudes in favour of delayed sexual debut were associated with lower sexual debut among both males (males: HR = 0.36, 95% CI = 0.25–0.52) and females (HR = 0.38, 95% CI = 0.25–0.57). Higher level of religiosity was associated with lower sexual debut rates only among females (HR = 0.59, 95% CI = 0.37–0.94).

**Conclusion:**

Given the increased risk for a number of sexually transmitted health problems, understanding the factors that are associated with premarital sexual debut will assist programmes in developing more effective risk prevention interventions.

## Background

No other single age group sustains the negative health outcomes from their sexual behaviours as do adolescents 10 to 19 years of age [1]. Adolescents in Nigeria have high burden of sexual and reproductive health problems [2]: the age-specific fertility rate of adolescent girls was 126 per 1,000 in 2003; and a quarter of females aged 15–19 years had begun childbearing [3]. Adolescent girls contribute 55% of all clandestine abortions in the country [4]; and the national HIV sero-prevalence figure for age 15–19 years is 3.6% [5].

Early onset of sexual intercourse is associated with increased lifetime prevalence of sexual partners, thereby increasing the risk exposure to sexually transmitted diseases, including HIV/AIDS, and pregnancy. Early sexual debut also increases the risk of HPV infection, due to cervical immaturity; and thus the risk of cervical cancer increases [6]. Additionally, given the risk of pregnancy, early sexual initiators are less likely to complete their schooling thereby limiting their social and vocational futures.

Empirical research, primarily from developed countries, indicates that the timing of sexual debut among adolescents is influenced by a wide range of factors including: age, gender, poverty, family structure, educational level, pubertal timing, socio-economic status, self-efficacy, peer influences, religiosity, knowledge and perceived risk of sexually transmitted infections, parenting practices and parental supervision, community, media and health inequalities [7,8].

Despite the high rates of early pregnancy and the associated sequelae relating to early sexual debut in sub-Saharan Africa, to date there has been very little research that has explored the determinants or predictors of the timing of sexual intercourse among adolescents. Where it does exist, as Blum and Mmari note [9], the majority of studies has focused on the relationships between premarital sexual initiation and socio-demographic variables but rarely go beyond that. The current study aims to address this research gap. Specifically, the aim of the study is to determine the predictors of age of sexual debut among a nationally representative sample of Nigerian adolescents.

## Methods

### Study Population and sampling

The data for this study was obtained as part of the 2005 National HIV/AIDS and Reproductive Health Survey (NARHS). NARHS is a nationally representative household survey of females (age 15–49 years) and males (age 15–64 years) of reproductive age. The primary objective of NARHS is to provide reproductive health information and data for monitoring the impact of reproductive health interventions.

The study population was randomly selected using a three level, multi-stage method that aimed at selecting eligible people in each of the 36 states in Nigeria with equal probability. Each state was first stratified by rural and urban areas, followed by random selection of enumeration areas, and subsequently individuals were selected at the household level. To increase the level of precision of the index obtainable at state level analysis, sample sizes in states with proportional allocation less than 250 were boosted to 250. Overall, the final sample allocated to each state was distributed proportionately by location (rural-urban) and gender.

A sample of 10,258 individuals was originally drawn, of whom 10,081 participated (1.7% non-response rate). No significant gender difference was noted in the non-response rate. A total of 2,423 adolescents (15–19 years old) were included in the NARHS sample, consisting of 1,258 males (51.9%) and 1,165 females (48.1%). Among the males, 96.6% were never married compared to 75.9% among females (p < 0.001). Never-married adolescents (age 15–19 years) are the focus of this study. Of the 2,099 never-married adolescents in the NARHS sample, 29 did not provide relevant or valid information on sexual experience or date of sexual debut and were excluded. Hence, the analytic sample for the study consisted of 2,070 never-married adolescents aged 15–19 years (1,195 males [57.7%] and 875 females [42.3%]).

### Data collection

Data were obtained through structured interview that covered various reproductive and sexual issues, including sexual behaviour, knowledge of HIV/AIDS and other sexually transmitted infections (STIs), condom perception and use, family planning (FP) attitudes and utilisation, gender-based violence, safe motherhood and communication. The questions were largely derived from UNAIDS general population HIV/AIDS indicator questionnaire [10] and the Nigeria Demographic Health Survey (NDHS) [3].

Questionnaires were primarily in English – the official language of the country – due to the linguistic complexity of Nigeria (with over 250 local languages); however, for each selected community, key words/phrases were translated into the local language(s). A similar approach was used successfully in the 2002 Youth Behavioural Surveillance Survey and the 2003 NARHS.

Data collection was carried out using trained interviewers, under the supervision of an independent research agency that worked closely with officials of State Ministry of Health and National Population Commission. Data from the questionnaires were originally entered into the computer using CSPro 2.6 with in-built check commands. The data were subsequently imported into SPSS. A system of monitoring and quality management was implemented using multi-disciplinary Technical Committee drawn from a broad group of stakeholders. Independent reviews of the entire survey process were undertaken by technical advisors from USAID (through MEASURE Evaluation), DFID and WHO.

The study received the approval of the Federal and the State Ministries of Health in Nigeria before implementation. Furthermore, the consent or assent of individual participants was obtained after being fully informed of the study purpose and procedures. Specifically, the questionnaire had a front page marked "confidentiality and consent", which was read to each respondent. Consents were freely obtained from each individual 15–17 years old but parental consent was not required. No name or other identifying information was maintained. Completed surveys were retrieved from interviewers daily by supervisors and centrally maintained until forwarded to the national coordination point immediately upon conclusion of the data collection.

### Measures

The dependent variable was age of first sex, while independent variables grouped into three categories – demographic, psychosocial, and community factors. Tables 1a and 1b show how each independent variable was defined and measured.

**Table 1:** 

**a:List of variables and how they were measured: demographic and community factors**		
**Variable**	**Items**	Measurement*

***Demographic factors***		
Age	Age at last birthday	Continuous variable
Sex (gender)	Sex of the respondent	Dichotomous (male, female)
Educational attainment	Did you attend any school?	Discrete: (none, primary,
	If yes, what is the highest level of school you attended?	secondary/higher)
Literacy in English language	What languages can you read with understanding?	Dichotomous (English, others)
Economic status	Type of dwelling structure	Discrete: Wealth index scale tertiles (high, middle, low)
	Which of these items do you have in your household? (list included household goods such as fridge, radio, television, fan, car, and selected agricultural assets such as cows and goats)	
	What is your main source of water for domestic use?	
	What is your main method for sewage disposal?	
***Community factors***		
Location	Rural or urban locality as designated by the National Population Commission	Dichotomous (rural, urban)
Region	State of resident of the respondent at the time of data collection, classified into one of the six national geo-political zones – North-Central, North East, North West, South East, South South, South West	Dichotomous (North, South)
Media exposure	How often do you listen to radio: every day, almost every day, at least once a week, less than once a week or not at all?	Dichotomous (every day or almost every day, others)
	How often do you watch television: every day, almost every day, at least once a week, less than once a week or not at all?	Dichotomous (every day or almost every day, others)

**b: List of variables and how they were measured: psychosocial factors**		

**Variable**	**Items**	Measurement*

***Psychosocial factors***		
Religiosity	How important is religion to you in helping you deal with problems?	Dichotomous (very important, others)
	How often do you attend religious congregational services?	Dichotomous (at least once a week, less than once weekly).
Alcohol use	During the last 4 weeks how often have you had drinks containing alcohol?	Dichotomous (had alcoholic drinks at least once, no drink at all)
Attitude toward premarital sex	Do you think that there is a need for young persons to delay the age at which they start sex?	Dichotomous (yes, no)
Family planning accessibility	Is (each of the following types of contraceptives – daily oral pills, after sex oral pills/emergency contraception, injectable contraceptives, IUD/Coil) affordable?	Scale
	Is (each of the following types of contraceptives – daily oral pills, after sex oral pills/emergency contraception, injectable contraceptives, IUD/Coil) easy to obtain?	
Family planning attitudes	Do you agree or disagree with the following statements:	Scale
	• Family planning (FP) encourages women to be promiscuous	
	• FP encourages young people to be loose	
	• Condoms encourage male infidelity	
• Use of FP can lead to infertility		
	• FP cause cancer or other diseases	
Condom norm	Do you think the following support or do not support young people using condoms to protect themselves from HIV and STIs if they are sexually active?	Scale
	• Relatives	
	• Community leaders	
	• Parents	
	• Friends	
	• Religious leaders	
	• Other young persons	
	• Health workers	
Condom efficacy	Do you agree or disagree with the following statements:	Scale
	• Male condoms protect against unplanned pregnancy	
	• Male condoms protect against the virus that causes AIDS	
	• Male condoms protect against diseases that are transmitted through sexual intercourse	
Condom access	• Would you say male condoms are affordable?	Scale
	• Do you agree or disagree that male condoms are easy to obtain?	
	• From which places or persons do you know of where you can obtain male condoms?	
HIV prevention knowledge	What can a person do to avoid getting the virus that causes AIDS?	Scale
	• Staying with one faithful uninfected partner	
	• Using condoms every time	
	• Abstaining from sex	
	• Delaying the onset of sexual intercourse	
	• Avoiding sex with commercial sex workers	
	• Reducing number of sexual partners	
	• Avoiding sex with people who have many sexual partners	
	• Avoid sharing of sharp objects like needles, razors	
Gender attitude	Is a husband justified in beating his wife in the following situations?	Scale
	• if she neglects the children	
	• if she argues with him	
	• if she goes out without telling husband	
	• if the food is not ready on time	
	• if he feels she is unfaithful	
	• if she refuses sex with husband	
Sexuality communication	How comfortable or uncomfortable would you feel talking about sexual matters to the following?	Scale
	• father	
	• mother	
	• brother	
	• sisters	
	• teachers	
	• religious leaders	

*Measure as used in the analysis, and not necessarily the form in the original dataset

Demographic factors consisted of age, gender, literacy in English language, education and economic status. "Wealth index" was constructed as proxy for economic status based on the method described by Filmer and Prittchett [11,12], which uses factor analysis to assign weights to specific household assets, type of dwelling structure and available utilities. Our sample was stratified into high, middle and low economic groups based on the frequency distribution of the overall scores.

Psychosocial factors measured included religiosity, alcohol use and personal attitude to premarital sex. Scales were constructed using exploratory factor analysis to assess other psychosocial factors: FP accessibility and attitudes; condoms perceived norms, efficacy and access; HIV prevention knowledge; gender attitude; and, sexuality-related communication (Table 2). Internal consistency and reliability of the scales was measured by Cronbach alpha, and the alpha model used is equivalent to the Kuder-Richardson 20 (KR20) coefficient for dichotomous data [13]. The community factors assessed were location (rural or urban), region (North or South) and media exposure.

**Table 2:** Measurement scales, Internal Consistency (Cronbach alpha) and Item Loadings

**Scale Name**	**Item content**	**Item loading**
Family planning accessibility (Cronbach alpha = .951)	Daily oral pills are affordable	.792
	After sex oral pills/emergency contraception are affordable	.787
	Injectables are affordable	.779
	IUD/Coil is affordable	.763
	Daily oral pills are easy to obtain	.762
	After sex oral pills/emergency contraception are easy to obtain	.735
	Injectables are easy to obtain	.735
	IUD/Coil is easy to obtain	.721
Family planning attitudes (Cronbach alpha = .846)	*FP encourages women to be promiscuous	.752
	*FP encourages young people to be loose	.723
	*Condoms encourage male infidelity	.695
	*Use of FP can lead to infertility	.578
	*FP cause cancer or other diseases	.465
Condom norm (Cronbach alpha = .882)	Relatives support young people using condoms to protect themselves from HIV and STIs if they are sexually active	.886
	Community leaders support young people using condoms to protect themselves from HIV and STIs if they are sexually active	.848
	Parents support young people using condoms to protect themselves from HIV and STIs if they are sexually active	.834
	Friends support young people using condoms to protect themselves from HIV and STIs if they are sexually active	.828
	Religious leaders support young people using condoms to protect themselves from HIV and STIs if they are sexually active	.802
	Other young persons support young people using condoms to protect themselves from HIV and STIs if they are sexually active	.802
	Health workers support young people using condoms to protect themselves from HIV and STIs if they are sexually active	.702
Condom efficacy (Cronbach alpha = .895)	Male condom protect against STI	.928
	Male condom protect against AIDS	.926
	Male condom protect against unplanned pregnancy	.832
Condom access (Cronbach alpha = .731)	Male condoms are easy to obtain	.833
	Male condoms are affordable	.798
	Know where to buy condom	.749
HIV prevention knowledge (Cronbach alpha = .774	HIV transmission can be prevented by avoiding sex with people who have many sexual partners	.797
	HIV transmission can be prevented by reducing number of sexual partners	.771
	HIV transmission can be prevented by avoiding sex with commercial sex workers	.743
	HIV transmission can be prevented by delaying onset of sexual intercourse	.622
	HIV transmission can be prevented by staying with one faithful uninfected partner.	.578
	HIV transmission can be prevented by avoiding the sharing of needles	.576
	HIV transmission can be prevented by abstaining from sex	.447
	HIV transmission can be prevented by using condoms every time	.420
Gender attitude (Cronbach alpha = .930)	*Beating wife is justified if she neglects the children	.804
	*Beating wife is justified if she argues with him	.787
	*Beating wife is justified if she goes out without telling husband	.778
	*Beating wife is justified if the food is not ready on time	.748
	*Beating wife is justified if he feels she is unfaithful	.716
	*Beating wife is justified if she refuses sex with husband	.664
Sexuality communication (Cronbach alpha = .825	Comfortable to discuss sexual matters with father	.755
	Comfortable to discuss sexual matters with mother	.741
	Comfortable to discuss sexual matters with brother	.656
	Comfortable to discuss sexual matters with sisters	.696
	Comfortable to discuss sexual matters with teachers	.736
	Comfortable to discuss sexual matters with religious leaders	.715

*Statements were reversed for scoring

### Data analysis

Data analysis was undertaken using SPSS (version 12.0) and STATA (version 9.0). Sampling weights, based on the sampling fractions derived from final sample size and the population of eligible respondents for the states was applied in the analysis. Univariate analysis was carried out to determine the proportion of sexually experienced adolescents, age of sexual debut and its median. Chi-square was used to assess gender differences in sexual experience pattern. Based on the result, subsequent data analyses were gender-disaggregated. Our analyses aimed, on the one hand, at determining factors significantly associated with sexual debut. On the other hand, it also aimed to examine, among the sexually experienced adolescents, factors that may predict early (before the age of 16 years) and later sexual initiation. Age 16 years was chosen because it represented the median obtained for sexual initiation. Chi-square (for categorical variables) and t-test (for continuous variables) were used to assess significance of bivariate associations.

Multivariate analysis was carried out using Cox proportional hazards regression (survival analysis). In building the model, backward likelihood ratio (stepwise) approach was used for exploratory purposes, and the result obtained from the process alongside theoretical knowledge from the literature with regards to factors associated with sexual behaviour among young people were used in selecting variables for the regression models. Two models were presented: the first included only selected demographic and community variables – factors that are likely to precede sexual initiation, while the second model included psychosocial factors in addition to the demographic and community factors. For each of the two models, hazard ratio (HR), 95% confidence interval (CI) and p-values were calculated separately for males and females. Literacy in English language and attendance frequency of attendance of religious services were not included in any of the models to avoid the problem of colinearity in view of their significant correlation with educational level and beliefs about the importance of religion respectively. With regards to the sexually experienced adolescents, given that very few variables were found to be significantly different between early and late sex initiators, survival analysis was not used to compare the two sub-groups. Statistical significance was set at an alpha level of 0.05.

## Results

Our final sample consisted of 1,195 males (57.7%) and 875 females (42.3%). A total of 1,661 (80.2%) reported never having had sexual intercourse (82.0% of males and 77.8% of females) (Table 3). For the sexually experienced individuals among our study population, the median age of sexual debut was slightly but not significantly lower for males (15 years) compared to females (16 years) (Mann-Whitney U test: p = 0.383).

**Table 3:** Distribution of respondents by age of sexual debut

**Age (years)**	**Male**		**Female**		**Total**	
	
	**Freq**	%	**Freq**	%	**Freq**	**%**
8	1	0.1	0	0.0	1	0
10	7	0.6	1	0.1	8	0.4
11	2	0.2	0	0.0	2	0.1
12	10	0.8	7	0.8	17	0.8
13	15	1.3	16	1.8	31	1.5
14	22	1.8	23	2.6	45	2.2
15	51	4.3	33	3.8	84	4.1
16	39	3.3	48	5.5	87	4.2
17	34	2.8	38	4.3	72	3.5
18	23	1.9	19	2.2	42	2.0
19	11	0.9	9	1.0	20	1.0
Never	980	82.0	681	77.8	1661	80.2

**Total**	**1195**	**100.0**	**875**	**100.0**	**2070**	**100.0**

Analysis of the data of the 2,070 never-married adolescents showed no gender difference with respect to sexual debut before age 16 (males 9.0%; females 9.1%; X^2 ^= 0.168 df = 2, p = 0.682). However, for older adolescents, females were statistically more likely to report sexual experience than males (13.0% vs. 9.0%; p < 0.001).

### Bivariate analyses

#### Males

Significant differences were found between the sexually experienced and abstinent groups with regards to English language literacy (p < 0.001), use of alcohol (p < 0.001), attitude toward premarital sex (p < 0.001), radio exposure (p = 0.002), television exposure (p = 0.006) and region (p < 0.001) (Table 4a). Significant differences were also obtained when males with no formal education were compared separately with those that had primary school (p = 0.009) and secondary/higher education groups (p < 0.001). Similarly, statistically significant difference obtained between primary school and secondary/higher educational group (p < 0.001). Compared to the low economic group, male adolescents in the middle (p = 0.019) and the high economic (p = 0.002) groups were significantly more likely to report ever having had sex. No statistical difference, however, was observed between the middle and high groups.

**Table 4:** 

**a: Association between selected factors and timing of sexual debut:*males***								
	**N**	**Never had sex (%)**	**Sex < 16 years (%)**	**Sex >16 years (%)**	**Never had sex vs. Ever had sex**		**Sex < 16 years vs. Sex ≥ 16 years**	

					**X**^2^	**p-value**	**X**^2^	**p-value**
***Educational level***								
No formal education	206	96.1	2.4^a^	1.5	6.727^a^	0.009	0.043^a^	0.835
Primary	232	89.7	6.0	4.3	20.522^b^	< 0.001	0.755^b^	0.385
Secondary or higher	756	75.9	11.8	12.3	41.931^c^	< 0.001	0.567^c^	0.451
*Total*	1194	82.1	9.0	8.9				
								
***Literacy in English***								
Yes	760	92.0	5.6	2.3				
No	427	76.2	11.1	12.8				
*Total*	1187	81.9	9.1	9.0	46.326	< 0.001	6.694	0.010
								
***Importance of religion***								
Very important	1082	82.5	8.4	9.1				
Others	85	80.0	11.8	8.2				
*Total*	1167	82.3	8.7	9.0	0.655	.418	0.711	0.399
								
***Frequency of religious attendance***								
None or less than once weekly	47	71.1	15.6	13.3				
At least once weekly	1148	82.4	8.8	8.8				
*Total*	1193	82.0	9.1	9.0	3.738	0.053	0.072	0.788
								
***Alcohol***								
Never	1054	86.2	7.9	5.9				
Ever	138	50.0	18.1	31.9				
*Total*	1192	82.0	9.1	8.9	111.363	< 0.001	8.255	0.004
								
***Young people should delay sexual debut***								
Yes	1064	84.8	7.3	7.9				
No	122	59.0	23.0	18.0				
*Total*	1186	82.1	8.9	8.9	47.405	< 0.001	0.942	0.332
								
***Radio exposure***								
Low	496	85.5	6.7	7.8				
High	611	78.7	11.1	10.1				
*Total*	1191	82.0	9.0	9.0	9.318	0.002	0.705	0.401
								
***Television exposure***								
Low	823	84.0	7.9	8.1				
High	366	77.6	11.5	10.9				
*Total*	1190	82.0	9.0	9.0	7.669	0.006	0.079	0.779
								
***Economic status***								
Low	330	87.3	5.5	7.3	5.458^a^	0.019	1.400^a^	0.237
Middle	470	81.1	10.2	8.7	0.856^b^	0.355	0.195^b^	0.659
High	396	78.5	10.9	10.6	9.621^c^	0.002	0.673^c^	0.412
*Total*	1196	81.9	9.1	8.9				
								
***Location***								
Rural	792	82.4	8.6	9.0				
Urban	404	80.9	10.1	8.9				
Total	1196	81.9	9.1	8.9	0.472	0.492	0.371	0.542
								
***Region***								
North	613	87.9	6.2	5.9				
South	581	75.7	12.0	12.2				
*Total*	1194	82.0	9.0	9.0	29.289	< 0.001	0.056	0.812

**b: Association between selected factors and timing of sexual debut: *females***								

	**N**	**Never had sex (%)**	**Sex < 16 years (%)**	**Sex >16 years (%)**	**Never had sex vs. Ever had sex**		**Sex < 16 years vs. Sex ≥ 16 years**	

					**X**^2^	**p-value**	**X**^2^	**p-value**

***Educational level***								
No formal education	117	89.7	6.8	3.4	0.336^a^	0.562	0.012^a^	0.913
Primary	135	87.4	8.1	4.4	11.567^b^	0.001	5.109^b^	0.024
Secondary or higher	622	73.6	9.6	16.7	14.086^c^	< 0.001	4.268^c^	0.039
*Total*	874	77.9	9.0	13.0				
								
***Literacy in English***								
Yes	594	85.3	7.3	7.3				
No	279	74.1	10.1	15.8				
*Total*	867	77.6	9.2	13.1	13.687	< 0.001	1.597	0.206
								
***Importance of religion***								
Very important	763	79.2	7.9	13.0				
Others	92	68.5	18.5	13.0				
*Total*	855	78.0	9.0	13.0	5.462	0.019	4.424	0.035
								
***Frequency of religious attendance***								
None or less than once weekly	81	86.4	8.6	4.9				
At least once weekly	792	77.0	9.1	13.9				
*Total*	893	77.9	9.0	13.1	2.997	0.083	2.487	0.115
								
***Alcohol***								
Never	818	78.6	8.8	12.6				
Ever	53	67.9	11.3	20.8				
*Total*	871	78.0	9.0	13.1	3.305	0.069	0.220	0.639
								
***Young people should delay sexual debut***								
Yes	783	80.6	7.5	11.9				
No	90	54.4	22.2	23.2				
*Total*	873	77.9	9.0	13.1	33.757	< 0.001	1.326	0.249
								
***Radio exposure***								
Low	547	79.0	10.2	10.8				
High	326	76.1	7.1	16.9				
*Total*	873	77.9	9.0	13.1	1.165	0.280	7.093	0.008
								
***Television exposure***								
Low	606	78.2	10.7	11.1				
High	269	76.6	5.6	17.8				
*Total*	875	77.7	9.1	13.1	0.289	0.591	11.401	0.001
								
***Economic status***								
Low	228	81.6	9.6	8.8	3.229^a^	0.069	1.422^a^	0.233
Middle	312	75.0	10.3	14.7	0.725^b^	0.395	0.850^b^	0.357
High	334	77.8	7.5	14.7	1.154^c^	0.283	3.845^c^	0.050
*Total*	874	77.8	9.0	13.2				
								
***Location***								
Rural	519	76.3	11.4	12.3				
Urban	355	80.3	5.6	14.1				
Total	874	77.9	9.0	13.0	1.860	0.173	6.942	0.008
								
***Region***								
North	367	86.9	5.2	7.9				
South	508	71.3	12.0	16.7				
*Total*	875	77.8	9.1	13.0	30.286	< 0.001	0.072	0.788

^a^No formal education vs. primary education/low social class vs. middle economic class.

^b^Primary vs. secondary & higher education/middle social class vs. high economic class.

^c^No formal education vs. secondary or higher education/low vs. high economic class

Sexually experienced males recorded statistically higher scores compared to abstinent males on the following scales: FP access (p < 0.001), FP attitudes (p < 0.001), condom norm (p < 0.001), condom efficacy (p < 0.001), condom access (p < 0.001) and sexuality communication (p = 0.008).

Comparing males with history of early and later sexual debut only literacy in English language (p = 0.010) and alcohol use (p = 0.004) differentiated the two groups.

#### Females

A higher proportion of female adolescents who were literate in English language were sexually abstinent (85.3%) compared to non-literate peers (74.1%) (p < 0.001) (Table 4b). Females who attached high level of importance to religion had statistically lower prevalence of sexual intercourse compared to others (79.2% vs. 68.5%) (p = 0.019). Girls with secondary school/higher education were significantly more likely to have initiated sex compared to non-educated girls (p < 0.001). However, those with primary school education were more likely to have remained sexually abstinent compared to those with secondary school/higher level of education (p = 0.001). Not surprisingly, personal attitudes in favour of delay of sexual debut were also significantly associated with less sexual engagement (p < 0.001). Also, there was a higher likelihood that females who reported having been sexually active came from the South compared with the North of Nigeria (p < 0.001). Economic status was not a significant predictor of sexual initiation.

Additionally, the sexually experienced group had significantly higher scores on the FP access (p = 0.014), FP attitudes (p < 0.001), condom norm (p < 0.001), condom efficacy (p < 0.001), condom access (p < 0.001), HIV prevention knowledge (p = 0.019) and gender attitude (p < 0.001) scales.

Comparison of females who initiated intercourse before and after 16 years of age showed significant differences only with regard to the following: importance attached to religion (p = 0.035); radio exposure (p = 0.008); television exposure (p = 0.008); secondary school/higher education versus no formal education (p = 0.039) and primary school (p = 0.024) groups; and, mean score on condom access scale (p = 0.039).

### Multivariate analyses

Of the four selected demographic and community factors – education, age, economic status, urban-rural location, and region – that were incorporated into the first model, only region was significantly associated with adolescent sexual initiation among both males and females. Additionally, educational attainment and age were also significantly associated with sexual initiation among the males (Table 5).

**Table 5:** Cox Proportional Hazards Regression Analysis by Gender: Selected Demographic and Community Factors

**Independent Variables**	**Males**			**Females**		
	
	**Hazard Ratio**	**95% C.I.**	**p-value**	**Hazard Ratio**	**95% C.I.**	**p-value**
*Educational level						
Primary	**3.02**	**1.36 – 6.71**	**0.01**	1.00	0.49 – 2.03	1.00
Secondary or higher	**5.43**	**2.58 – 11.44**	** < 0.001**	1.32	0.71 – 2.44	0.38
Age	**1.16**	**1.04 – 1.29**	**0.01**	1.09	0.96 – 1.23	0.19
**Economic status						
Middle class	0.99	0.69 – 1.44	0.97	0.90	0.61 – 1.31	0.58
High class	0.97	0.62 – 1.50	0.88	0.79	0.49 – 1.27	0.33
Urban location	0.77	0.55 – 1.07	0.12	0.85	0.58 – 1.25	0.41
Southern region	**1.44**	**1.07 – 1.95**	**0.02**	1.71	**1.22 – 2.40**	**0.01**

*Referent group was those with no formal education.

**Referent group was those in the low economic class.

As the Cox proportionate hazards model incorporating demographic, community and psychosocial factors shows in Table 6, factors associated with early sexual debut among both males and females were more positive attitudes regarding condom efficacy (males: HR = 1.28, 95% CI = 1.07–1.53; females: HR = 1.24, 95% CI = 1.05–1.46) and more positive family planning attitudes (males: HR = 1.19, 95% CI = 1.09–1.31; females: HR = 1.18, 95% CI = 1.07–1.30). Conversely, adolescents who favoured delay in sexual debut were about two and half times less likely to initiate sexual intercourse compared with their peers (males: HR = 0.36, 95% CI = 0.25–0.52; females: HR = 0.38, 95% CI = 0.25–0.57).

**Table 6:** Cox Proportional Hazards Regression Analysis by Gender: Demographic, Community, and Psycho-social Factors

**Independent Variables**	**Males**			**Females**		
	
	**Hazard Ratio**	**95% C.I.**	**p-value**	**Hazard Ratio**	**95% C.I.**	**p-value**
***Demographic factors***						
*Educational level						
Primary	2.16	0.81 – 5.77	0.12	1.31	0.44 – 2.91	0.78
Secondary or higher	2.32	0.91 – 5.92	0.08	1.08	0.49 – 2.39	0.85
Age	1.00	0.89 – 1.13	0.97	0.95	0.82 – 1.10	0.48
Stronger opinion on importance of religion	1.07	0.63 – 1.82	0.81	**0.59**	**0.37 – 0.94**	**0.03**
**Economic Status						
Middle class	1.01	0.67 – 1.52	0.96	0.83	0.53 – 1.30	0.40
High class	0.89	0.54 – 1.50	0.66	0.67	0.38 – 1.18	0.17
						
***Psycho-social factors***						
Use of alcohol	**1.90**	**1.38 – 2.62**	** < 0.001**	0.90	0.51 – 1.60	0.71
Supportive attitude to adolescent delay of sexual debut	**0.36**	**0.25 – 0.52**	** < 0.001**	**0.38**	**0.25 – 0.57**	** < 0.001**
Perceived higher FP access	1.03	0.97 – 1.09	0.29	1.02	0.95 – 1.09	0.58
More positive attitudes to family planning	**1.19**	**1.09 – 1.31**	** < 0.001**	**1.18**	**1.07 – 1.30**	**0.01**
More positive about community support for condom use	1.05	0.99 – 1.12	0.13	1.02	0.95 – 1.09	0.675
More positive about condom efficacy	**1.28**	**1.07 – 1.53**	**0.01**	**1.24**	**1.05 – 1.46**	**0.01**
Higher condom access	**1.42**	**1.14 – 1.76**	**0.01**	1.18	0.96 – 1.45	0.11
Gender attitude	0.95	0.89 – 1.02	0.15	**1.13**	**1.04 – 1.23**	**0.01**
HIV prevention knowledge	0.90	0.79 – 1.02	0.09	1.08	0.96 – 1.22	0.19
Sexuality communication	0.97	0.90 – 1.06	0.53	1.00	0.90 – 1.10	0.93
						
***Community factors***						
Higher radio exposure	0.90	0.65 – 1.23	0.49	0.92	0.64 – 1.31	0.64
Higher television exposure	1.29	0.87 – 1.91	0.21	0.99	0.64 – 1.52	0.96
Urban location	0.70	0.49 – 1.01	0.57	0.90	0.59 – 1.36	0.60
Southern region	1.20	0.87 – 1.65	0.27	1.38	0.93 – 2.05	0.11

*Referent group was those with no formal education.

**Referent group was those in the low economic class

In addition, females who attached more importance to the value of religion were significantly less likely to start sex during adolescence (HR = 0.59, 95% CI = 0.37–0.94) while those that had more positive gender-related disposition, which reflects personal disposition to females' social status and freedom from gender-related violence, were found to have a slightly but statistically significantly increased risk of sexual initiation (HR = 1.13, 95% CI = 1.04–1.23). Among males, the use of alcohol was found to be significantly associated with sexual initiation during adolescence (p < 0.001): those who reported drinking were about twice as likely to report having had sexual intercourse (HR = 1.90, 95% CI = 1.38–2.62). Perceived higher access to condom was also significantly associated with sexual debut among never-married male adolescents (HR = 1.42, 95% CI = 1.14–1.76) (Table 6).

## Discussion

This study had examined the demographic, psychosocial and community factors associated with adolescent sexual initiation using nationally representative sample of Nigerian adolescents. While a number of studies have been reported on adolescent sexual behaviour in Nigeria, most were small-scale studies that disallow national generalisations.

Overall, 18.0% of male and 22.2% of female never-married adolescents (15–19 years) reported being sexually experienced in our study; figures that are consistent with other Nigerian studies [16-21]. The NDHS, with its national coverage, provides a more reliable portrait of adolescent sexual behaviours than small-scale reports. Our figure for females 15 to 19 years old who were sexually active (22.2%) is much lower than the retrospective report of 20–24 year olds in the NDHS 75.4% of whom reported having had sex before the age of 20. That figure, however, failed to disaggregate the ever from never married youth. As reported by the NDHS, 56% of young people 20–24 years were married by the age of 20 years. Thus, married adolescents accounted for a large proportion of the sexually active females. Consistent with our findings, using the 2003 NDHS data and focusing on never-married females, Isiugo-Abanihe and Oyediran [22] reported that 20.9% of females aged 15–19 years had ever had sexual intercourse. With regards to males, our figure (18%) is, on the whole, comparable to that of 2003 NDHS, which reported 27.4% of males aged 20–24 years as having had sex by the age of 18 years and 12.7% of males as being married by age of 20. NDHS' figure of 7.9% for the proportion of males that had had sex by age 15 also compares well with our figure of 9.1% for the sub-group.

Based on secondary analysis of Demographic and Health Survey (DHS) data, Mensch and colleagues have reported that for West Africa the proportions of 20–24 years who had engaged in premarital sex were: 18.7% for Gabon (2000 DHS), 21.1% for Ivory Coast (1998 DHS), 22.0% for Ghana (2003 DHS), 24.7% for Benin (2001 DHS) [23]. Given the difference in age cohort and time difference between the periods when the various surveys were conducted, our findings on Nigerian female sexual engagement is comparable to that of female youth in many neighboring countries but significantly lower than the rates in many Francophone countries: Cameroon (35.2%; 2004 DHS), Burkina Faso (42.5%; 2003 DHS), Chad (62.5%; 1996–97 DHS), Mali (53.5%; 2001 DHS), and Niger (74.5%; 1998 DHS). The proportion of Nigerian adolescents engaged in sex is also lower than that found in many developed countries of Europe and North America [24,25].

The higher prevalence of females engaged in sex in our study agrees with the observation of the United States National Academies of Science that more females compared to males report having had sex by age 18 in sub-Saharan Africa, while in Latin America and the Caribbean it is the reverse [26]. The present finding also questions the assertion by some that females consistently underreport and males over report their sexual activities. Likewise, an experimental study on data collection methods among unmarried adolescents in Kenya did not uncover evidence of female adolescent underreporting in face-to-face interviews when compared with the use of audio-assisted self-interview [27].

Our finding regarding a comparatively lower tendency of females with higher levels of religiosity to initiate sex during adolescence is consistent with reports of previous studies [28,29]. As different from the findings for females, we did not find statistically significant relationship between religiosity and sexual behaviour among the males. This too is consistent with the research of Rostosky and colleagues who concluded, in their review of ten published works, that while religiosity delays the sexual debut of adolescent females, the results are mixed for adolescent males [30]. Halpern et al explored the relative impact of testosterone and religiosity on adolescent sexual behaviour and concluded that testosterone overrode religiosity [31].

Consistent with previous reports [32], personal attitude favouring delayed sexual debut was associated with avoiding sexual intercourse among both males and females. The theory of planned behaviour holds that attitudes constitute one of the determinants of health behaviour [33].

The use of alcohol, which was not significantly associated with sexual intercourse for females, was significantly associated with sex among males in the survival analysis model with psychosocial variables. It is important to note that the proxy used in the measurement of alcohol-related practices in this study was "alcohol use in the last four weeks prior to the study", which may or may not actually reflect the use of alcohol prior to sexual debut. However, noting that association between sexual engagement and alcohol use has been reported in numerous adolescent studies in different parts of the world including in the United States [34], the Caribbean [35], and Japan [36] whereas very little focus has so far been given to such relationship in the Nigerian environment, we considered it worthwhile to explore the relationship despite the potential weakness of our measure. Our finding in this dimension should be regarded as exploratory. The repeated observations of the association between adolescent sexual initiation and alcohol use are in agreement with Jessor's cluster of risk behaviours among adolescents [37] which, according to his problem behaviour theory [38], can be traced to a common underlying factor of unconventionality – the tendency to transgress social norms. Conventionality-unconventionality has been conceptualised as a dimension underlying and summarising an orientation towards, commitment to, and involvement in the prevailing values, standards of behaviour, and established institutions of the adult society [38]. In general, greater conventionality is associated with greater involvement in health-maintaining behaviours whereas greater unconventionality relates to less involvement in health-maintaining behaviour and greater involvement in health-risky behaviours.

Our findings also indicate that both males and females who have stronger belief in the efficacy of condoms and more positive attitudes to FP as well as males more convinced of their ability to access condoms were more likely to initiate sex during adolescence. While the association sounds logical, caution is needed since the cross-sectional nature of the study precludes deducing causality. Confidence that condoms will prevent unwanted pregnancy and STI can remove the "fear" element that may have inhibited a young person to engage in premarital sex. As the Extended Parallel Process Model [39] hypothesises and empirical studies have shown [40], the element of fear plays an important role in the decision-making about sex. Likewise, an adolescent's conviction of his ability to get condom when he needs it, particularly when convinced of its efficacy, may have a disinhibiting effect on sexual behaviour. On the other hand, it is equally possible that sexual debut precedes the condom-related attitude we measured. Sexually active adolescents may have sought more information on the sources for and effectiveness of condoms and through first-hand experience prove its efficacy and their ability to access condom. Studies have shown that improved access to condoms can lead to more condom acquisition by high-school adolescents though may not necessarily lead to increases in either sexual activity or condom use [41].

When looking at the relationships between attitudes toward gender equity and sexual debut in adolescence we found that Nigerian female adolescents who reported more positive attitudes toward gender equality and less tolerance of gender-based violence were also more likely than peers to report having had premarital sex. Perhaps these findings are also consistent with Jessor's *unconventionality *hypothesis on the clustering of risk behaviours. Maybe it reflects a more liberal attitude which is also reflected in more openness to sexual initiation. Further research is needed to answer this question.

The present analyses showed significant regional difference in the proportion of sexually active males (12.1% north, 24.3% south; X^2^= 29.289, p < 0.001) and females (13.1% north, 28.7% south; X^2^= 30.286, p < 0.001) at bivariate level as well at multivariate level when only selected socio-demographic factors were included in the model (males: HR = 1.44, 95% CI = 1.07–1.9; females HR = 51.7; 95% CI = 11.22–2.40). However, region was not a significant independent factor when psychosocial and other factors were added to the model. Thus, it is likely but uncertain that factors other than geography distinguished the more conservative and largely Islamic North from the South with her more educated, more urban and largely Christian population. The difference recorded between the North and the South in the present study does not reflect *per se *actual differences in proportion of sexually active adolescents; rather, the differences are among those who initiate intercourse in premarital context. As the NDHS shows, the practice of adolescent marriage is far more prevalent in the North compared to the South [3]. Thus, a major difference between the regions regarding adolescent sexual engagement is the context of sexual activity: mostly intra-marital in the North and predominantly pre-marital in the South.

Among sexually experienced adolescents, our comparison of early and later sexual debutants surprisingly identified only few statistically significant factors at bivariate level. Literacy in English language and alcohol use were significant factors among males while some levels of education, religiosity and media exposure were significant factors among females. The reasons for this finding are not clear, but one possibility is that the power of the study to detect the differences between the two groups is low. The issue deserves further study.

One of the challenges to studying sexual behaviour is the question of the validity of the data due to recall and social desirability biases. By focusing on age 15–19 years, the possibility of recall bias, which has been noted to increase with age [42], is limited in our study. Limiting the analysis to the 15–19 age group also makes the picture more current in terms of associating timing of sexual debut and potential predictive factors. Our use of survival analysis made for adequate allowance for age censoring and avoids a common error in studies of age at first sex among adolescents [43].

A major limitation of our study, however, is that it is cross-sectional, which necessarily limits causal conclusions. In addition, some of the psychosocial factors may not have actually preceded sexual initiation. To address the limitation relating to the uncertainty of the temporal relationship between the independent variables, particularly the psychosocial factors, and sexual initiation, we presented two different survival analysis models. Whereas one excluded the psychosocial and other factors that may have uncertain temporal relationship, the other incorporated all the factors of interest in the study. With the difference in the factors identified between the two models, there is a need for further study about antecedents and predictors of adolescent sexual initiation among Nigerian adolescents using longitudinal approach.

Our interest in this study is the never-married adolescents, and we recognise that this category may be substantially different from that of the married adolescent with regards to several contextual as well as sexual behavioural factors. Early marrying adolescents may, for example, be more likely to have an early premarital sexual debut and lesser education and, thus, their exclusion poses a challenge in terms of external validity of the results. The result of our study may not be generalised to all adolescents in Nigeria particularly with the fairly high rate of marriage among adolescent females and the associated geographic diversity within the national context.

Nevertheless, our findings have significant implications for adolescent sexual and reproductive interventions in Nigeria since abstinence still constitutes a primary pillar in the prevention of HIV, other sexually transmitted infections and teenage unwanted pregnancy. Among other strategies, health education and behaviour change communication programmes targeting young people in Nigeria need to consider how the issues of myths, wrong information, and poor attitude to such simple but critical interventions such as condom use impact behaviour. Additionally, the role of religiosity (though only found for females) raises the question of the potential influence of the faith community in promoting adolescent sexual health.

## Conclusion

A fifth of Nigerian adolescents initiate sexual intercourse prior to marriage. While this rate do not reach the levels found in Europe or North America, the special risks of HIV infection and unwanted pregnancy in sub-Saharan Africa make this an especially vulnerable population. There is, therefore, the need to ensure effective programmes that will reach those who are most vulnerable. To be effective such programmes must be built upon the understanding of those factors associated with an increased likelihood for early sexual debut.

## Competing interests

The authors declare that they have no competing interests.

## Authors' contributions

AOF participated in the acquisition of the data and conceptualisation of the study, performed statistical analysis, and drafted the manuscript; RWB participated in the conceptualisation of the study, reviewed data analysis results and critically revised the manuscript. Both authors read and approved the final manuscript.

## Pre-publication history

The pre-publication history for this paper can be accessed here:



## References

[B1] Bearinger LH, Sieving RE, Ferhuson J, Sharma V (2007). Global perspectives on the sexual and reproductive health of adolescents: patterns, prevention, and potential. Lancet.

[B2] Fatusi AO (2005). Adolescent sexual and reproductive health needs in Nigeria: shaping a pragmatic and effective response. J Comm Med & Pry Health Care.

[B3] National Population Commission (NPC) [Nigeria] and ORC Macro (2004). Nigeria Demographic and Health Survey 2003.

[B4] Bankole A, Oye-Adeniran BA, Singh S, Adewole IF, Wulf O, Sedgh G, Hussain R (2006). Unwanted pregnancy and induced abortion in Nigeria: causes and consequences.

[B5] Federal Ministry of Health (Nigeria) (2005). National HIV/Syphilis sero-prevalence sentinel survey among pregnant women attending antenatal clinics in Nigeria Technical report Abuja, National AIDS/STI Control Programme (NASCP), Federal Ministry of Health.

[B6] Ludicke F, Stalberg A, Vassilakos P, Major AL, Campana A (2001). High- and intermediate-risk human papillomavirus infection in sexually active adolescent females. J Pediatr Adolesc Gynecol.

[B7] Lammers C, Ireland M, Resnick M, Blum R (2000). Influences on adolescents' decision to postpone sexual intercourse: a survival analysis of virginity among youths aged 13 to 18 years. J Adolesc Health.

[B8] Santelli JS, Kaiser J, Hirsch L, Radosh A, Simkin L, Middlestadt S (2004). Initiation of sexual intercourse among middle school adolescents: the influence of psychosocial factors. J Adolesc Health.

[B9] Blum RW, Mmari KN (2006). Risk and protective factors affecting adolescent reproductive health in developing countries.

[B10] UNAIDS HIV/AIDS Prevention indicator survey. Knowledge, attitudes, sexual behaviour, stigma. Individual questionnaire. http://www.emro.who.int/GFATM/guide/tools/unaidspopulation/individual.pdf.

[B11] Filmer D, Pritchett L (1998). Estimating wealth effects without expenditure data – or tears: with an application to educational enrollments in States of India. Policy Research Working paper 1994.

[B12] World Bank Measuring living standards: household consumption and wealth indices. Quantitative techniques for health equity analysis – Technical Note No 4.

[B13] SPSS Inc (2001). Reliability analysis statistics. SPSS release 1101 help file.

[B14] Sun G, Shook TL, Kay GL (1996). Inappropriate use of bivariate analysis to screen risk factors for use in multivariable analysis. J Clin Epidemiol.

[B15] Harrell FE, Lee KL, Mark DB (1996). Multivariable prognostic models: issues in developing models, evaluating assumptions and adequacy, and measuring and reducing errors. Stat Med.

[B16] Dare OO, Oladepo O, Cleland JG, Badru OB (2001). Reproductive health needs of young persons in markets and motor parks in south west Nigeria. Afr J Med Med Sci.

[B17] Otoide VO, Oronsaye F, Okonofua FE (2001). Sexual behaviour and contraceptive use among secondary school adolescents in Benin City, Nigeria. J Obstet Gyaecol.

[B18] Slap GB, Lot L, Huang B, Daniyam CA, Zink TM, Succop PA (2003). Sexual behaviour of adolescents in Nigeria: cross sectional survey of secondary school students. BMJ.

[B19] Ajuwon AJ, Olaleye A, Faromoju B, Ladipo O (2006). Sexual behavior and experience of sexual coercion among secondary school students in three states in North Eastern Nigeria. BMC Public Health.

[B20] Wagbatsoma VA, Okojie OH (2006). Knowledge of HIV/AIDS and sexual practices among adolescents in Benin City, Nigeria. Afr J Reprod Health.

[B21] Amoran OE, Onadeko MO, Adeniyi JD (2005). Parental influence on adolescent sexual initiation practices in Ibadan, Nigeria. Int Q Community Health Educ.

[B22] Isiugo-Abanihe UC, Oyediran KA (2004). Household socio-economic status and sexual behaviour among Nigerian female youth. Afr Popul Stud.

[B23] Mensch B, Grant MJ, Blanc AK (2006). The changing context of sexual initiation in sub-Saharan Africa. Popul Dev Rev.

[B24] Lloyd CB, (ed) (2005). National Research Council and Institute of Medicine. Growing up global: the changing transitions to adulthood in developing countries Panel on Transitions to Adulthood in Developing Countries Committee on Population and Board on Children, Youth and Families Division of Behavioural and Social Sciences and Education.

[B25] Singh S, Darroch JE, Frost JJ (2001). Socioeconomic disadvantage and adolescent women's sexual and reproductive behavior: the case of five developed countries. Fam Plann Perspect.

[B26] Wellings K, Collumbien M, Slaymaker E, Singh S, Hodges Z, Patel D, Bajos N (2006). Sexual behaviour in context: a global perspective. Lancet.

[B27] Mensch BS, Hewet PC, Erulkar AS (2003). The reporting of sensitive behaviour by adolescents: a methodological experiment in Kenya. Demography.

[B28] Nonnemaker JM, McNeely CA, Blum RW (2003). Public and private domains of religiosity and adolescent health risk behaviors: evidence from the national longitudinal study of adolescent health. Soc Sci Med.

[B29] Rew L, Wong YJ (2006). A systematic review of associations among religiosity/spirituality and adolescent health attitudes and behaviors. J Adolesc Health.

[B30] Rostosky SS, Wilcox BL, Wright MLU, Randall BA (2004). The impact of religiosity on adolescent sexual behaviour: a review of the evidence. J Adoles Res.

[B31] Halpern CT, Udry JR, Campbell B, Suchindran C, Mason GA (1994). Testosterone and religiosity as predictors of sexual attitudes and activity among adolescent males: a biosocial model. J Biosoc Sci.

[B32] Paradise JE, Cote J, Minsky S, Lourenco A, Howland J (2001). Personal values and sexual decision-making among virginal and sexually experienced urban adolescent girls. J Adolesc Health.

[B33] Godin K, Kok G (1996). The theory of planned behaviour: a review of its applications to health-related behaviors. Am J Health Promot.

[B34] Weden MM, Zabin LS (2005). Gender and ethnic differences in the co-occurrence of adolescent risk behaviors. Ethn Health.

[B35] Ohene SA, Ireland M, Blum RW (2005). The clustering of risk behaviors among Caribbean youth. Matern Child Health J.

[B36] Takakura M, Wake N, Kobayashi M (2007). Relationship of condom use with other sexual risk behaviors among selected Japanese adolescents. J Adolesc Health.

[B37] Jessor R (1991). Risk behavior in adolescence: a psychosocial framework for understanding and action. J Adolesc Health.

[B38] JE Donovan, R Jessor, FM Costa (1991). Adolescent health behavior and conventionality-unconventionality: an extension of problem-behavior theory. Health Psychol.

[B39] Gore TD, Bracken CC (2005). Testing the theoretical design of a health risk message: reexamining the major tenets of the extended parallel process model. Health Educ Behav.

[B40] Witte K, Murray L, Hubbell AP, Liu WY, Sampson J, Morrison K (2001). Addressing cultural orientation in fear appeals: promoting AIDS-protective behaviors among Mexican immigrants and African-American adolescents and American and Taiwanese college students. J Health Commun.

[B41] Kirby D, Brener ND, Brown NL, Peterfreund N, Hillard P, Harrist R (1999). The impact of condom distribution in Seattle schools on sexual behavior and condom use. Am J Public Health.

[B42] Gupta N, Mahy M (2003). Sexual initiation among adolescent girls and boys: trends and differentials in sub-Saharan Africa. Arch Sex Behav.

[B43] Zaba B, Pisani E, Slaymaker E, Ties Boerma J (2004). Age at first sex: understanding recent trends in African demographic surveys. Sex Transm Inf.

